# Evidence that cyanobacterial Sll1217 functions analogously to PGRL1 in enhancing PGR5-dependent cyclic electron flow

**DOI:** 10.1038/s41467-019-13223-0

**Published:** 2019-11-22

**Authors:** Marcel Dann, Dario Leister

**Affiliations:** Plant Molecular Biology, Faculty of Biology, Ludwig-Maximilians University Munich, Großhaderner Str. 2, D-82152 Planegg-Martinsried, Germany

**Keywords:** Organelles, Photosynthesis

## Abstract

In plants and cyanobacteria, the PGR5 protein contributes to cyclic electron flow around photosystem I. In plants, PGR5 interacts with PGRL1 during cyclic electron flow, but cyanobacteria appear to lack PGRL1 proteins. We have heterologously expressed the PGR5 and PGRL1 proteins from the plant *Arabidopsis* in various genetic backgrounds in the cyanobacterium *Synechocystis*. Our results show that plant PGR5 suffices to re-establish cyanobacterial cyclic electron flow (CEF), albeit less efficiently than the cyanobacterial PGR5 or the plant PGR5 and PGRL1 proteins together. A mutation that inactivates *Arabidopsis* PGR5 destabilises the protein in *Synechocystis*. Furthermore, the *Synechocystis* protein Sll1217, which exhibits weak sequence similarity with PGRL1, physically interacts with both plant and cyanobacterial PGR5 proteins, and stimulates CEF in *Synechocystis*. Therefore, Sll1217 partially acts as a PGRL1 analogue, the mode of action of PGR5 and PGRL1/Sll1217 proteins is similar in cyanobacteria and plants, and PGRL1 could have evolved from a cyanobacterial ancestor.

## Abstract (web summary)

In *Arabidopsis*, PGR5 and PGRL1 heterodimers enable cyclic electron flow to safeguard photosystem I during high light intensity. Here, the authors provide evidence that cyanobacteria, while lacking an obvious PGRL1 homologue, use an analogous PGRL1-like protein to facilitate cyclic electron flow.

## Introduction

Two general modes of photosynthetic electron transport are employed by oxygenic photosynthetic mechanisms: linear and cyclic electron flow (LEF and CEF). LEF is driven by photosystems I (PSI) and II (PSII), generating ATP and NADPH. CEF generates ATP only, and is driven by PSI^1–4^ Different environmental and metabolic conditions require appropriate adjustment of the ATP/NADPH ratio. Therefore, rates of LEF and CEF need to be balanced accordingly. Two principal routes have been described for CEF, involving electron transport from ferredoxin via two different photosynthetic complexes “back” to the plastoquinone pool. The “antimycin A insensitive” pathway^5^ is utilised by cyanobacteria^6–8^, C_4_ plants^9,10^ and, under low light conditions^11^, also by C_3_ plants, and involves transport of electrons from ferredoxin to the NADH dehydrogenase-like complex (NDH)^12,13^. On the other hand, the antimycin A sensitive pathway involves the thylakoid proteins PROTON GRADIENT REGULATION 5 (PGR5)^14^ and the subsequently identified PGR5-LIKE PHOTOSYNTHETIC PHENOTYPE 1 (PGRL1)^15^, and is therefore also known as “PGR5-dependent CEF”. The PGR5-dependent CEF contributes to the acidification of the thylakoid lumen particularly under high light intensities^16^ and it operates to safeguard PSI function^17^ in the C_3_ plant *Arabidopsis thaliana*. In the green alga *Chlamydomonas reinhardtii*, PGR5-dependent CEF becomes relevant during anoxia, high light or when carbon fixation is limited^18^.

In *A. thaliana*, PGRL1 and PGR5 form a heterodimer which is thought to shuttle electrons from PSI via ferredoxin to the cytochrome *b*_*6*_*f* complex^15,19^, and inactivation of either protein results in reduced thylakoid lumen acidification^15^. Cyanobacteria possess an antimycin A sensitive CEF pathway that involves a protein with clear homology to PGR5 (Ssr2016 or synPGR5)^20^, but they lack an obvious homologue of PGRL1^21^. This work examines how synPGR5 mediates CEF in the apparent absence of a PGRL1 protein, and addresses the question of the evolutionary origin of PGRL1. We found that plant PGRL1 has a functional counterpart in cyanobacteria, which has only weak sequence similarity with PGRL1 and cannot be replaced by PGRL1. However, the plant PGRL1-PGR5 module can functionally replace the cyanobacterial module comprising synPGR5 and the *Synechocystis* counterpart of PGRL1, indicating that no additional plant-specific proteins are required to drive CEF in *Synechocystis*. Therefore, plant PGRL1 could actually derive from a cyanobacterial progenitor.

## Results

### Plant PGR5 and PGRL1 can restore CEF in *Synechocystis*

Inactivation of the *synPGR5* gene in the cyanobacterium *Synechocystis* sp. PCC6803 impairs CEF^20^. In the *synpgr5* background (see Methods), we expressed the *A. thaliana* genes for PGR5 (*atPGR5*) and PGRL1A (*atPGRL1*), either separately or together, under the transcriptional control of the strong *psbA2* promoter^22,23^. We also expressed *synPGR5* and *atPGR5*_*G130S*_ (the mutated atPGR5 found in the *A. thaliana pgr5-1* mutant, in which the abundance and activity of the protein is dramatically reduced owing to a non-synonymous point mutation; Supplementary Fig. 1)^14,24^ in the *synpgr5* background.

**Fig. 1:**
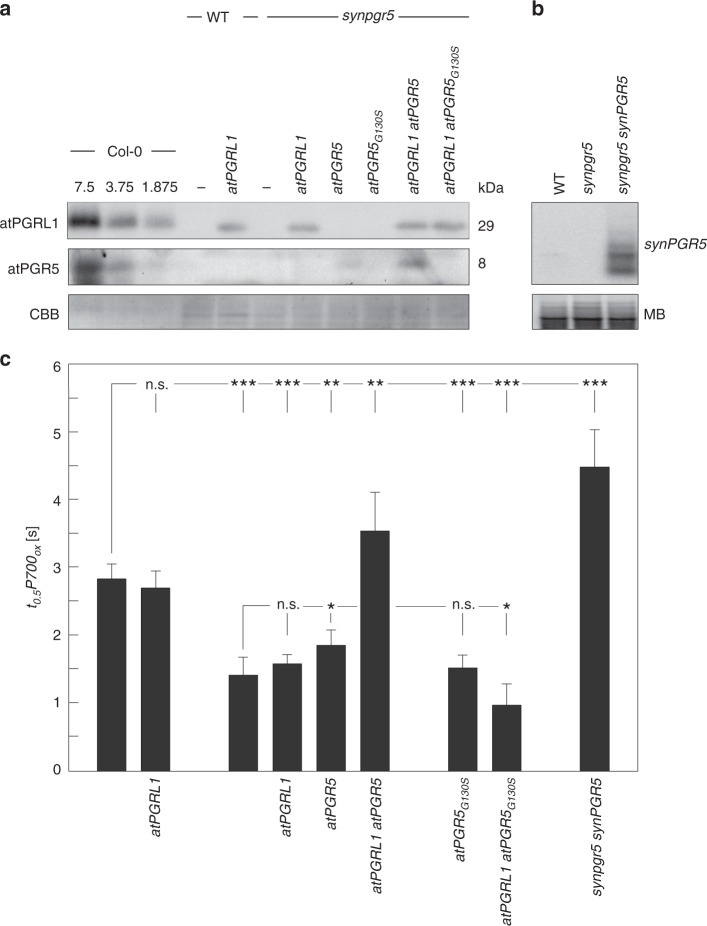
*Arabidopsis* PGR5 and PGRL1 can restore cyclic electron flow in the *Synechocystis synpgr5* mutant. **a** Immunoblot detection of atPGRL1 and atPGR5 proteins in *Synechocystis* strains expressing the two plant proteins either separately or together in the WT or *synpgr5* background. WT and *synpgr5* strains, as well as different dilutions of protein extracts from WT *A. thaliana* (Col-0), were employed as controls. Col-0 extracts were loaded in different dilutions corresponding to 7.5, 3.75, and 1.875 µg of thylakoid protein, and 30-µg aliquots of *Synechocystis* thylakoid proteins were loaded per lane. Staining with Coomassie Brillant Blue served as loading control. **b** Transcripts of *synPGR5* accumulate to much higher levels in the *synpgr5 synPGR5* strain than in the WT, as determined by Northern analysis using a *synPGR5*-specific probe. 20 µg of total RNA were loaded per lane. Methylene blue (MB) staining served as loading control. **c** The bar chart shows mean *t*_*0.5*_*P700*_*ox*_ values (see Methods), as a proxy for the level of CEF (see text for details), for *Synechocystis* transformants expressing plant PGR5 and/or PGRL1 or overexpressing cyanobacterial PGR5 (synPGR5), together with the appropriate controls. Error bars correspond to the standard deviation for *n* = 6/6/6/4/6/3/7/16 (order as displayed) independent experiments, and statistically significant differences with respect to WT or *synpgr5* according to Holm-corrected two-sided Student’s *t*-tests are indicated by asterisks (^*^P ≤ 0.05, ^**^P ≤ 0.01, ^***^P ≤ 0.001, n.s., not statistically significant).

The resulting *Synechocystis* strains were then characterised with respect to the steady-state concentrations of the proteins encoded by the introduced genes (Fig. 1a, b) and the rate of PSI oxidation, as quantified by the rate constant *t*_*0.5*_*P700*_*ox*_ (see Methods)—the time required for half-maximum oxidation of the reaction centre (P700) of PSI upon exposure to far-red (FR) light illumination^25^. A high *t*_*0.5*_*P700*_*ox*_ value therefore indicates slow PSI oxidation, and can be taken as an indirect measure of high CEF, as the oxidation of P700 upon exposure to FR light following depletion of respiratory donors by prolonged dark incubation is primarily mediated by CEF^26,27^ (Fig. 1c, Supplementary Fig. 2). In the transformants, atPGRL1 and atPGR5 were successfully expressed. But atPGR5_G130S_ failed to accumulate in detectable amounts, suggesting that—as in plants—the mutant protein is unstable in cyanobacteria (Fig. 1a). Moreover, expression of atPGRL1 promoted the accumulation of atPGR5, whereas the presence of atPGR5 had no effect on atPGRL1 levels. Thus—as in plants^15,18,24^—atPGRL1 stabilises atPGR5 in *Synechocystis*, but not vice versa. Because our atPGR5-specific antibody^14^ did not recognise synPGR5, we monitored the expression of cyanobacterial PGR5 in the *synPGR5 synpgr5* line by Northern blot analysis (Fig. 1b). Absence of synPGR5 impairs CEF in *Synechocystis*^20^, and we indeed detected a significant decrease in *t*_*0.5*_*P700*_*ox*_ in *synpgr5* compared to wild type (WT) *Synechocystis* (Fig. 1c). Accordingly, in the synPGR5 overexpressor (*synPGR5 synpgr5*), increased *t*_*0.5*_*P700*_*ox*_ values relative to WT were measured, suggesting that CEF is enhanced by the increased synPGR5 dosage driven by the strong *psbA2* promoter. Similarly, co-expression of atPGR5 and atPGRL1 in the *synpgr5* background restored *t*_*0.5*_*P700*_*ox*_ values to WT-like levels, implying that plant PGR5 and PGRL1 are sufficient to mediate CEF in this background. However, when the two plant proteins were separately expressed in the *synpgr5* host, atPGR5, but not atPGRL1, significantly enhanced *t*_*0.5*_*P700*_*ox*_ values. Moreover, CEF levels in *atPGR5 synpgr5* were lower than in either WT *Synechocystis* or the synPGR5 overexpressor strain, indicating that atPGR5 can only partially replace synPGR5. In contrast, expression of atPGRL1 in the presence of synPGR5 fails to boost CEF, as demonstrated by the fact that *t*_*0.5*_*P700*_*ox*_ values in WT *Synechocystis* remain essentially unaltered upon expression of atPGRL1. As expected, the mutated atPGR5 had no effect.

A plausible explanation for these results is that both PGR5 and PGRL1 have functional counterparts in cyanobacteria, and we designate the putative *Synechocystis* PGRL1 pendant as “*Synechocystis* PGRL1-LIKE” or “synPGRL1-LIKE”. Apparently, cyanobacterial CEF is fully compatible with the plant PGRL1-PGR5 system, since co-expression of PGRL1 and PGR5 fully restores CEF in the *Synechocystis* mutant *synpgr5*. In contrast, limited compatibility exists between the individual components and their respective partners from the other species. Of the two hybrid plant/cyanobacterial systems tested here—atPGR5-synPGRL1-LIKE (in *synpgr5 atPGR5*) and atPGRL1-synPGR5 (in WT *Synechocystis* expressing atPGRL1)—the latter is not functional, and atPGR5-synPGRL1-LIKE exhibits lower CEF activity than synPGR5-synPGRL1-LIKE (in WT *Synechocystis*). The incompatibility of atPGRL1 with cyanobacterial PGR5 is in accordance with the absence of any obvious homologue of PGRL1 in cyanobacterial genomes.

### The *Synechocystis* protein Sll1217 acts as a PGRL1 analogue

**Fig. 2:**
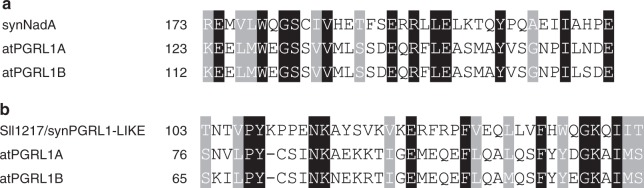
Short stretches of the cyanobacterial proteins synNadA and Sll1217/synPGRL1-LIKE share sequence similarity with PGRL1. Original CyanoBase^42^ pBlast hits (see Supplementary Fig. 3) for synNadA and Sll1217/synPGRL1-LIKE were obtained when only the N-terminal segment (between the cTP and the first TM region) of atPGRL1A was used as the query. Here, alignments of the PGRL1-like sequence stretch of synNadA (**a**) and Sll1217/synPGRL1-LIKE (**b**) with the corresponding stretches in atPGRL1A and atPGRL1B, reconstructed using MUSCLE^43^, are shown.

**Fig. 3:**
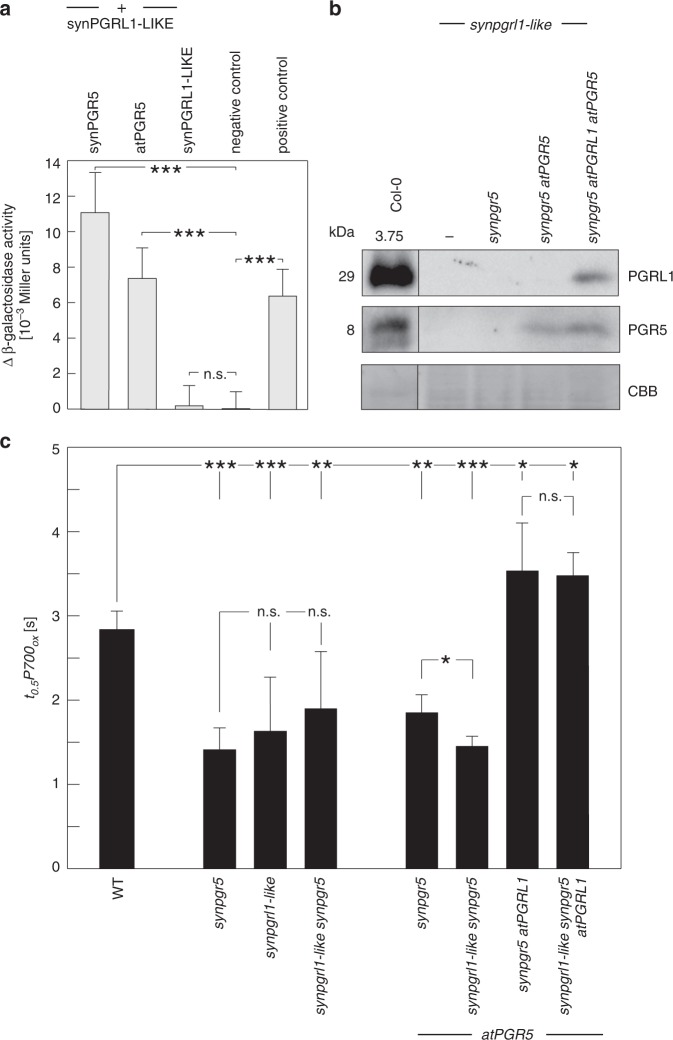
Cyanobacterial PGRL1-LIKE is functionally analogous to plant PGRL1. **a** Bacterial two-hybrid analyses. The degree of interaction is indicated by differential β-galactosidase activity (i.e. activity above the baseline activity of the negative control). Known interacting proteins served as positive control, known noninteracting proteins as negative control (see Methods). Error bars correspond to the standard deviation for *n* = 3 independent experiments and statistically significant differences with respect to WT or *synpgr5* according to Holm-corrected two-sided Student’s *t*-tests are indicated with asterisks (^***^P ≤ 0.001, n.s., not statistically significant). **b** Immunoblot detection of atPGRL1 and atPGR5 proteins in various strains of *Synechocystis*, all of which lack Sll1217/synPGRL1-LIKE (*synpgrl1-like*) and/or synPGR5. Extracts of the *synpgrl1-like* strain and WT *A. thaliana* were used as functional controls, and a segment of the Coomassie Brilliant Blue (CBB) stained blot is provided as a loading control. **c** Effects of the absence of synPGRL1-LIKE on CEF. Average *t*_*0.5*_*P700*_*ox*_ values are provided for the *Synechocystis* strains from panel **b**, as well as other strains from Fig. 1 as controls. Error bars correspond to the standard deviation for *n* = 6/6/5/5/4/3/6/4 (order as displayed) independent experiments and statistically significant differences according to Holm-corrected two-sided Student’s *t*-tests are indicated with asterisks (^*^P ≤ 0.05, ^**^P *≤* 0.01, ^***^P ≤ 0.001, n.s., not statistically significant).

To identify a functional counterpart of PGRL1 in cyanobacteria, we searched for *Synechocystis* proteins that display limited sequence similarity to PGRL1 (see Methods). Although cyanobacterial genomes including *Synechocystis* do not contain genes for true PGRL1 homologues^21^, we found two proteins containing short sequence stretches with limited sequence similarity to PGRL1 (Fig. 2, Supplementary Fig. 3). Of these, synNadA/Sll0622 (318 amino acids) is annotated as a quinolinic acid synthetase that catalyses a step in the de novo biosynthesis of NAD^28,29^. Because of this well-described function in bacterial primary metabolism, which is also reflected by the marked level of protein sequence conservation between species (e.g. 41% identity at 95% query coverage [*E* = 1E^−^^79^] between synNadA and its *E. coli* homologue), we consider an additional role of synNadA in CEF to be unlikely. In agreement with this assessment, synNadA did not interact with synPGR5 in bacterial 2-two-hybrid (B2H) assays and lack of synNadA did not decrease *t*_*0.5*_*P700*_*ox*_ values (Supplementary Fig. 4), effectively disqualifying it as PGRL1 analogue. The other candidate, Sll1217 (225 aa), has few known homologues outside cyanobacteria (e.g. *Pseudomonas spec*. and planctomycetes), and contains a 40-amino-acid stretch with sequence similarity to PGRL1 (Fig. 2, Supplementary Fig. 3). Unlike PGRL1, it lacks transmembrane domains and contains only three cysteine residues (Supplementary Fig. 3), in contrast to six conserved cysteine residues in plant PGRL1 proteins^19^. Sll1217 exhibits overall similarity to uracil-DNA glycosylases (UDGs), which play a role in DNA repair^30^. Sll1217 is therefore classified as member of the UDG-like superfamily—more specifically, the UDG4 family. However, to our knowledge, close homologues of Sll1217 have escaped genetic or biochemical characterisation so far, rendering its molecular function effectively unknown. B2H interaction analysis showed that Sll1217 can interact with synPGR5 and, to a slightly lesser extent, with atPGR5 (Fig. 3a, Supplementary Fig. 5a). To further test whether Sll1217 could represent synPGRL1-LIKE, we generated a sll1217 knockout strain (*sll1217*) and introduced this mutation into various genetic backgrounds, obtaining *sll1217 synpgr5*, *sll1217 synpgr5 atPGR5*, and *sll1217 synpgr5 atPGR5 atPGRL1* strains. Immunoblot analysis indicated that absence of Sll1217 had no effect on the accumulation of atPGR5 or atPGR5 + atPGRL1 in the latter two strains (Fig. 3b). However, *t*_*0.5*_*P700*_*ox*_ analysis implied that lack of Sll1217 indeed has an impact on CEF activity (Fig. 3c, Supplementary Fig. 5b). In fact, compared with WT *Synechocystis*, in the *sll1217* strain, CEF activity (as indicated by decreased *t*_*0.5*_*P700*_*ox*_ values) is reduced to virtually the same level as in the *synpgr5* mutant, suggesting that Sll1217 represents the predicted synPGRL1-LIKE, serving the same function for synPGR5 as PGRL1 performs for plant PGR5. With respect to its functional interactions with the plant proteins, in the *synpgr5 atPGR5* background, the concomitant absence of Sll1217/synPGRL1-LIKE decreased CEF back to *synpgr5* levels, whereas in the *synpgr5 atPGR5 atPGRL1* background loss of Sll1217/synPGRL1-LIKE had no marked additional effect (Fig. 3c, Supplementary Fig. 5b).

In sum, this implies that Sll1217/synPGRL1-LIKE functionally interacts with plant PGR5 in mediating CEF in *Synechocystis*.

## Discussion

Taken together, the results presented in this study strongly suggest that both plant PGR5 and PGRL1 have functional counterparts in cyanobacteria, synPGR5, and synPGRL1-LIKE. Moreover, the marked effect of synPGR5 overexpression indicates that the capacity for PGR5-mediated CEF in *Synechocystis* is greater than suggested by experiments with cyanobacterial strains that lack flavodiiron proteins^31^. While the PGR5 proteins are conserved and plant PGR5 can partially substitute for its cyanobacterial homologue, synPGRL1-LIKE displays only weak sequence similarity to PGRL1 and cannot be replaced by plant PGRL1. However, the plant PGRL1-PGR5 module can functionally replace the synPGRL1-LIKE-synPGR5 module in the *synpgr5 synpgrl1-like* background, indicating that no additional plant-specific proteins are required to drive CEF in *Synechocystis*. We therefore conclude that the mechanisms used by PGR5 and PGRL1/synPGRL1-LIKE proteins are analogous in cyanobacteria and plants. Plant PGRL1 might actually derive from synPGRL1-LIKE, possibly involving fusions of synPGRL1-LIKE with other genes. Since synPGRL1-LIKE lacks transmembrane domains (in contrast to PGRL1), and the full set of cysteines conserved among plant PGRL1 proteins, it seems likely that the synPGR5-synPGRL1-LIKE module alone is not sufficient to mediate CEF in cyanobacteria, but that additional, as yet unidentified factors are required. These hypothetical factor(s), possibly mediating thylakoid membrane association and supplying additional cysteines for redox regulation, might then be sufficient to replace, together with synPGR5 and synPGRL1-LIKE, the PGR5 and PGRL1 proteins in plants.

## Methods

### *Synechocystis* strains and their generation

*Synechocystis sp*. PCC6803 mutant strains were generated by homologous recombination following transformation with non-replicative plasmids derived from pICH69822 (E. Weber; Icon Genetics GmbH, Halle, Germany). Molecular cloning/plasmid construction was done using Golden Gate^32^, Gibson assembly^33^, and Q5^®^ site-directed mutagenesis (NEB). Knockouts of ssr2016 (*synpgr5*), sll0622 (*synnada*) and sll1217 (*synpgrl1-like*) were generated by transformation with pΔsm2, pΔsll0622, and pΔsll1217 constructs (each of which bears 500-700-bp segments of 5′ and 3′ genomic DNA sequence flanking a KanR and a SpecR cassette, respectively) that result in deletion of the corresponding ORFs (Supplementary Figs. 6, 7 and 8). (Over-)expressors of *synPGR5*, *atPGRLA,* and/or *atPGR5* were generated by transformation with p6xHis-SM2/pSM2-6xHis, pP1, pP5, or pP15 constructs, respectively (Supplementary Fig. 9). Each construct carries 900-bp segments of 5′ and 3′ genomic DNA sequence derived from the designated *Synechocystis* neutral site slr0168 flanking synthetic genes expressing mature atPGRL1A and/or atPGR5 proteins (i.e. lacking their respective cTPs as predicted by ChloroP v1.1^34^ under the control of the *Synechocysis psbA2* promoter. In addition, each harbours a CmR-sacB double selection cassette (DSC) allowing for marker-less gene replacement^35^, enabling the expression cassettes to be inserted into the slr0168 ORF. Segregation was achieved by successive rounds of selection on increasing concentrations of chloramphenicol, and was confirmed by PCR. Marker-less gene replacement by deletion of the DSC was subsequently achieved by negative selection on 5% sucrose following one cultivation cycle of segregated mutants on antibiotic-free medium, and was confirmed by PCR and immunodetection of the product of the reconstituted *atPGRL1A* gene. Primer sequences are provided in Supplementary Table 1.

### Nucleic acid analysis

Cloning PCRs were performed with Q5^®^ high-fidelity polymerase (NEB), and genotyping PCRs were performed using the Phire Plant Direct PCR Kit (ThermoFisher Scientific). Knockout constructs were confirmed by restriction analysis, and expression constructs were further subjected to Sanger sequencing analysis. Point mutations in atPGR5 expression strains were verified by Sanger sequencing of PCR amplicons. Total RNA was extracted from photoautotrophically grown cultures^36^ and RNA concentrations were determined spectroscopically using a Nanodrop 2000 spectrophotometer (Peqlab). Northern blot transcript analysis was performed^37^ using 20-µg aliquots of total RNA and the full-length [α-^32^P]CTP-labelled ssr2016 coding sequence (CDS) as the probe.

### Protein extraction and detection

Thylakoid proteins were prepared^38^, denatured, and fractionated by SDS PAGE on 4%/10% polyacrylamide Tris-Tricine gels^39^, and transferred onto PVDF membrane (Millipore Immobilon-P Transfer Membrane, 0.45 µm) overnight by capillary transfer with 1 × PBS (0.137 M NaCl, 0.0027 M KCl, 0.01 M Na_2_HPO_4_, 0.0018 M KH_2_PO_4_, pH 7.4). Proteins were fixed by methanol treatment and subsequent drying. After reactivation with methanol, PVDF membranes were equilibrated with TBST (25 mM Tris-HCl, 0.15 M NaCl, 0.05% Tween-20, pH 7.5) at 4 °C and blocked with 3% BSA (w/v) in TBST for 2 h. Exposure to the primary antibody was performed overnight at 4 °C with gentle shaking (50 rpm horizontal), using rabbit antibodies (1:10,000 dilution) raised against atPGRL1^15^ and atPGR5^14^. Secondary antibody (HRP-conjugated anti-rabbit, 1:10,000 dilution; Agrisera) decoration was performed for 3 h at 4 °C at 50 rpm. Chemiluminescence signal detection was performed using Pierce^™^ ECL Western Blotting Substrate (ThermoFisher Scientific) and the ChemiDoc^™^ MP Imaging System (BioRad).

### Bacterial two-hybrid analysis

Protein-protein interactions were determined with the BACTH (Bacterial Adenylate Cyclase Two-Hybrid System) kit (Euromedex). In this assay, the strength of interactions between pairs of proteins is determined as differential β-galactosidase activity (i.e. activity above the baseline activity of the negative control) and measured as *o*-nitrophenyl-β-galactoside cleavage. Non-interacting protein pairs (the putative transcription factor Sll0149 and bacterioferritin-associated ferredoxin Bfd/Ssl2250) served as negative controls, whereas the bacterioferritin Bfr2 (Slr1890)-Bfd combination, two proteins with known transient interaction in *P. aeruginosa*^40^, was employed as the positive control.

### Sequence analysis

Homology searches by local protein sequence alignment (pBlast) were conducted using the implementations in the NCBI^41^ and CyanoBase^42^ versions. The CyanoBase pBlast expectation value threshold *E* was altered to 10^4^ to allow for a less stringent alignment search. Local and global alignments shown in Fig. 2 and Supplementary Figs. 1 and 3 were generated by MUSCLE^43^, as implemented in MEGA v.10.0.4^44^, using default settings.

### P700 oxidation/absorbance analysis

The kinetics of oxidation of the PSI reaction centre were analysed by measuring P700 absorbance with the DUAL-PAM-100 instrument (Walz). *Synechocystis* cultures were grown photoautotrophically to late exponential phase (OD ~0.6–0.9) in shaken flasks (120 rpm) of BG11-based media supplemented with the appropriate antibiotics (kanamycin, 10 µg ml^−1^; spectinomycin, 10 µg ml^−1^; chloramphenicol 6.5 µg ml^−1^) at 30 °C under a continuous light flux of 30 µE m^−2^ s^−1^. Cells were harvested by a 4-min centrifugation at 2800 *g* and 25 °C, washed twice in BG11, and adjusted to OD_730 nm_ = 5. Cell suspensions were aliquoted into 2-ml fractions and incubated in the dark overnight (≥16 h) at 25 °C and 120 rpm. Oxidation of P700 under 38 µE far-red light (DUAL-PAM-100 FR intensity 3; *λ* = 720 nm) was monitored by changes in absorbance at 820 nm relative to 870 nm. Curves were normalised by equating the absorbance baseline (average absorbance over 3 s of dark before onset of FR) to 0, and equating the absorbance maximum during 60 s FR illumination to one. Rate constants (time required to reach 50% P700 oxidation) were acquired from normalised curves by extracting the time points at which Δabs exceeded 0.5.

### Reporting summary

Further information on research design is available in the Nature Research Reporting Summary linked to this article.

## Supplementary information

Supplementary Information

Reporting Summary

## Data Availability

The authors declare that all data presented in this study are available within the figures and its Supplementary Information file. The source data underlying Figs. 1 and 3, and Supplementary Figs 3 and 4, as well as detailed corresponding statistics, are provided as a Source Data file. Other data that support the study are available from the corresponding author upon reasonable request.

## Source data

Source Data
