# Supplementary material for: Evidence that cyanobacterial Sll1217 functions analogously to PGRL1 in enhancing PGR5-dependent cyclic electron flow
Source: Nat Commun. 2019 Nov 22;10:5299. doi: 10.1038/s41467-019-13223-0 (PMC6876563; doi:10.1038/s41467-019-13223-0)
Supplement: Supplementary file 1 — Supplementary Information [file 41467_2019_13223_MOESM1_ESM.pdf]

Supplementary Information

Evidence that cyanobacterial Sll1217 functions analogously to PGRL1 in enhancing PGR5-dependent cyclic electron flow

Dann & Leister

Nature Communications, 2019

```
synPGR5   1  --------MFAPIVILVRQQLGKAKFNQIRGKAIALHCQTITNFCNRVGIDAKQRQNLIR
atPGR5   61  KNVNEGKGLFAPLVVVTRNLVGKKRFNQLRGKAIALHSQVITNFCNRVGIDAKQRQNLIR
                                                     ↑
                                               pgr5-2 (S98F)

synPPG5  53  LAKSNGKTLGLLA
atPGR5  121  LAKKNGERLGFLA
                      ↑
               pgr5-1 (G130S)
```

Supplementary Figure 1 PGR5 protein alignment and positions of mutation in atPGR5. The sequence of the mature atPGR5 protein[1] was aligned with synPGR5 (Ssr2016) using MUSCLE[2]. Identical/similar amino acids are highlighted by black/grey shading. The positions of published non-synonymous mutations in atPGR5 are annotated relative to start codon in the precursor protein with cTP. The homology between the two proteins is 45%/56% identity/similarity at the amino acid level; it increases to 65%/80% when only the region from aa 69 to aa 133 of atPGR5 is considered.

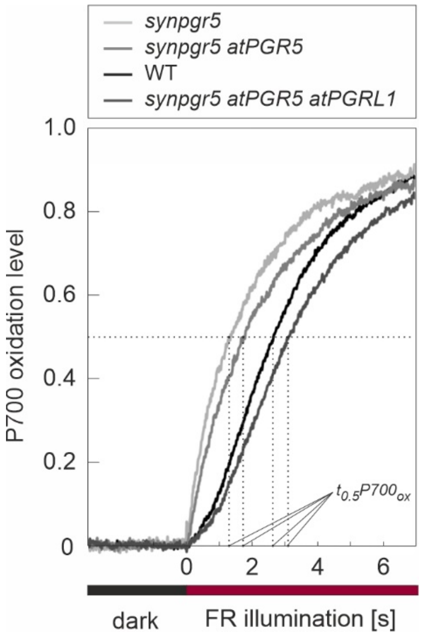

Supplementary Figure 2 Determination of $t_{0.5}P700_{ox}$ for representative *Synechocystis* strains. Dark-incubated cultures (normalized to equal $OD_{730\ nm}$ values) were subjected to 3 s of dark/measuring light illumination to determine $P700^+$ baseline absorbance, followed by 60 s of FR illumination to induce P700 oxidation and reach steady-state oxidation levels. Baseline absorbance was set to 0, while absorbance maxima were set to 1, respectively. For each measurement, the rate constant for maximum P700 oxidation was determined as the time point ($t_{0.5}P700_{ox}$) at which the baseline corrected/normalized oxidation curves exceeded 0.5 for the first time. Mean values of $t_{0.5}P700_{ox}$ and standard deviations thereof were determined for all genotypes and employed as a proxy for CEF activity (see text).

a

```
>sll0622 quinolinate synthetase
          Length = 318

 Score = 28.1 bits (61), Expect = 0.65,   Method: Compositional matrix adjust.
 Identities = 12/38 (31%), Positives = 20/38 (52%)

Query: 123 KEELMWEGSSVVMLSSDEQRFLEASMAYVSGNPILNDE 160
           +E ++W+GS +V  +  E+R LE    Y     I + E
Sbjct: 173 REMVLWQGSCIVHETFSERRLLELKTQYPQAEIIAHPE 210
```

b

```
>sll1217 unknown protein
          Length = 225

 Score = 25.8 bits (55), Expect = 1.1,   Method: Compositional matrix adjust.
 Identities = 13/41 (31%), Positives = 23/41 (56%), Gaps = 1/41 (2%)

Query: 16  SNVLPYCSI-NKAEKKTIGEMEQEFLQALQSFYYDGKAIMS 55
            +N +PY    NKA    + E  + F++ L  F++ GK I++
Sbjct: 103 TNTVPYKPPENKAYSVKVKERFRPFVEQLLVFHWQGKQIIT 143
```

c

```
synNADA     1  ------------MFTAVAPPQETLPRDLVGAIQSLKKELNAVILAHYYQEAAIQDIADY-
atPGRL1A   61  ATTEQSGPVGGDNVDSNVLPYCSINKAEKKTIGEMEQEFLQALQSFYYDGKAIMSNEEFD
atPGRL1B   50  ASTDQSGQVGGEEVDSKILPYCSINKNEKRTIGEMEQEFLQAMQSFYYEGKAIMSNEEFD

synNADA    48  -LGDSL---GLSQQAASTDADVIVFAGVHFMAETAKILNP--HKLVLLPDLEAGCSLADS
atPGRL1A  122  NLKEELMWEGSSVVMLSSDEQRFLEASMAYVSGNPILNDEEYDKLKLKLKIDGSDIVSEG
atPGRL1B  111  NLKEELMWEGSSVVMLSSDEQRFLEASMAYVSGNPILSDEEYDKLKMKLKMDGSEIVCEG

synNADA   102  --CPPREFAEFKQRHPDHLVISYINCTAEIKALSDIIC---TSSNAVKIVQQLPPDQKII
atPGRL1A  182  PRCSLRSKKVYSDLAVDYFKMLLLNVPATVVALGLFFFLDDITGFEITYIMELPEPYSFI
atPGRL1B  171  PRCSLRSKKVYSDLAIDYFKMFLLNVPATVVALGLFFFLDDITGFEITYLLELPEPFSFI

synNADA   157  FAPDRNLGRYVMEQTGREMVLWQGS--CIVHETFSERRLLELKTQYPQAEIIAHPECEKA
atPGRL1A  242  FT-------------------WFAAVPVIVYLALSITKLI-----IKDFLILKGP-CPNC
atPGRL1B  231  FT-------------------WFAAVPAIVYLALSLTKLI-----LKDFLILKGP-CPNC

synNADA   215  ILRHADFIGSTTALLNYSGKSQGKEFIVGTEPGIIHQMEKLSPSKQFIPLPNNSNCD---
atPGRL1A  277  GTENTSFFGT------------------------------------ILSISSGGKTNTVK
atPGRL1B  266  GTENVSFFGT------------------------------------ILSIPNDSNTNNVK

synNADA   272  CNECPYMRLNTLEKLYWAMQRRSPEITLPEATMAAALKPIQRMLAMS
atPGRL1A  301  CTNC------GTAMVY---DSGSRLITLPEGSQA-------------
atPGRL1B  290  CSGC------GTEMVY---DSGSRLITLPEGGKA-------------
```

d

```
Sll1217      1   -MSELQTLIRTIRQEAEREPF-PLDSPIYEQAGK------DALDPILFGG----------
atPGRL1A    61   ATTEQSGPVGGDNVDSNVLPYCSINKAEKKTIGEMEQEFLQALQSFYYDGKAIMSNEEFD
atPGRL1B    50   ASTDQSGQVGGEEVDSKILPYCSINKNEKRTIGEMEQEFLQAMQSFYYEGKAIMSNEEFD

Sll1217     43   NLGSQLCFFGRD---LGADEVRQGQPLIG--AAGRLVRKGFFEAWQGRVPRGQDDLQTVC
atPGRL1A   121   NLKEELMWEGSSVVMLSSDEQRFLEASMAYVSGNPILNDEEYDKLKLKLKIDGSDIVSEG
atPGRL1B   110   NLKEELMWEGSSVVMLSSDEQRFLEASMAYVSGNPILSDEEYDKLKMKLKMDGSEIVCEG

Sll1217     98   QRILLTNTVPYKPPENKAYSVKVKERFRPFVEQLLVFHWQ---GKQIITLGT--EAFK--
atPGRL1A   181   PRCSLRSKKVYSDLAVDYFKMLLLNVPATVVALGLFFFLDDITGFEITYIMELPEPYSFI
atPGRL1B   170   PRCSLRSKKVYSDLAIDYFKMFLLNVPATVVALGLFFFLDDITGFEITYLLELPEPFSFI

Sll1217    151   --WFAP-----YAPKGQLDEFFQGGDRYECSLDVLIKAKTAAGKGSQKIV---RLMPLPH
atPGRL1A   241   FTWFAAVPVIVYLALSITKLIIK---------DFLILKGPCPNCGTENTSFFGTILSISS
atPGRL1B   230   FTWFAAVPAIVYLALSLTKLILK---------DFLILKGPCPNCGTENVSFFGTILSIPN

Sll1217    201   PSPLNKRYYGQFPTML----QRRLTEIAF----
atPGRL1A   292   GGKTNTVKCTNCGTAMVYDSGSRLITLPEGSQA
atPGRL1B   281   DSNTNNVKCSGCGTEMVYDSGSRLITLPEGGKA
```

Supplementary Figure 3 Short stretches of Sll0622/synNadA and Sll1217/synPGRL1-LIKE share limited sequence similarity with PGRL1.

a, b Original CyanoBase[3] pblast hits for synNadA and Sll1217/synPGRL1-LIKE were obtained using either the complete sequence of the mature atPGRL1A protein or its N-terminal region (between cTP and first TM region) only. **c, d** Alignments of full-length Sll0622 and Sll1217 and mature PGRL1 proteins from *Arabidopsis thaliana* (without cTPs) generated using MUSCLE[2]. The Sll0622 protein shares 13.8%/ 26.8% and 15.0%/27.7% identity/similarity at the amino-acid level with atPGRL1A and atPGRL1B, respectively. The Sll1217 protein shares 13%/29% and 14%/28% identity/similarity at the amino-acid level with atPGRL1A and atPGRL1B, respectively. Conserved cysteine residues in PGRL1 proteins[4] are highlighted in green, the three cysteine residues present in Sll1217 are highlighted in cyan.

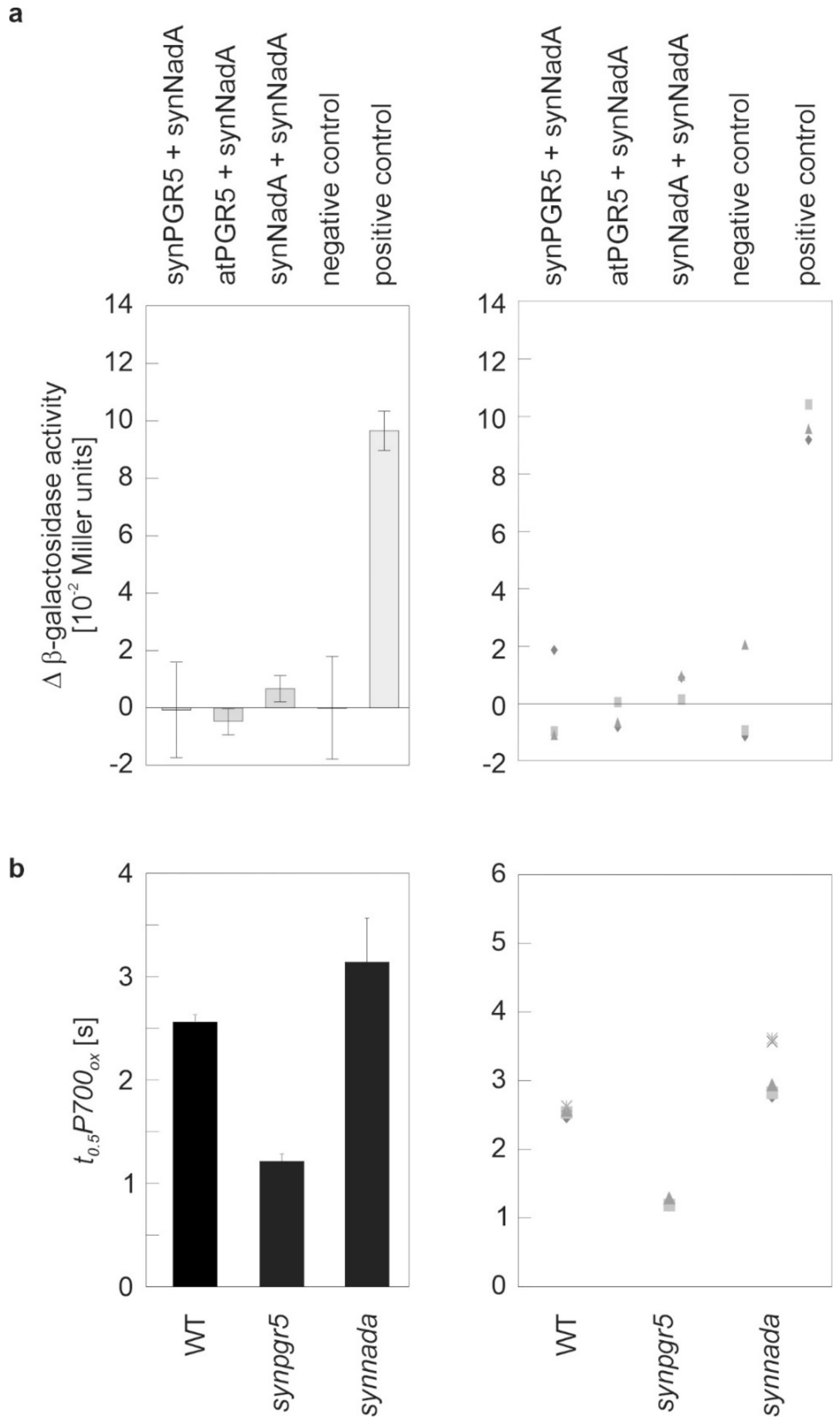

Supplementary Figure 4 The synNadA protein is not a functional PGRL1 analogue.
a The synPGR5 and synNadA proteins do not physically interact. Bacterial two-hybrid analyses were performed as described in Fig. 3a. **b** Lack of synNadA does not affect CEF, as determined by P700 oxidation measurements. Data are provided as averages with standard deviations (left) and dot plots (right). Sample sizes *n* were 3 for B2H assays (**a**) and 5/3/5 for $t_{0.5}P700_{ox}$ of WT, *synpgr5* and *synnada* (**b**).

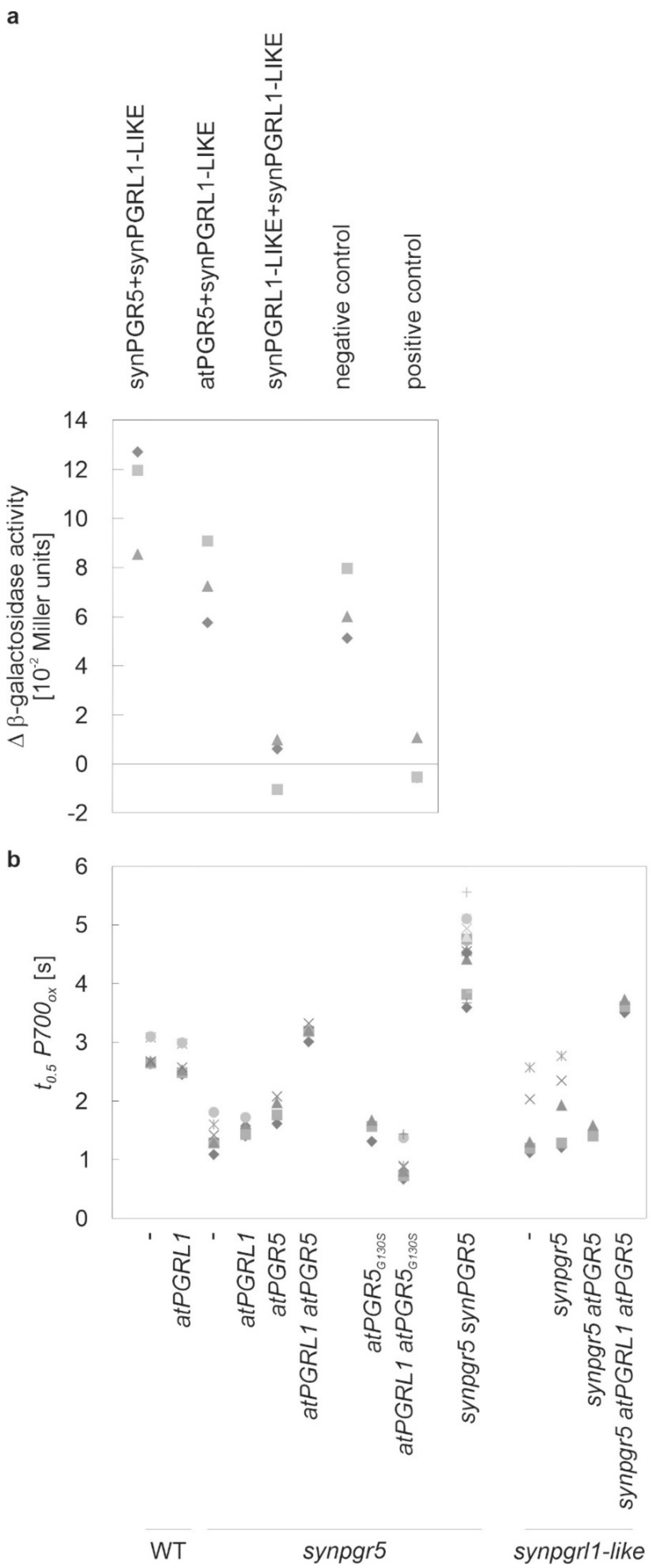

Supplementary Figure 5 Dot plots for the data shown in Fig. 3a (**a**) and Fig. 3c (**b**).

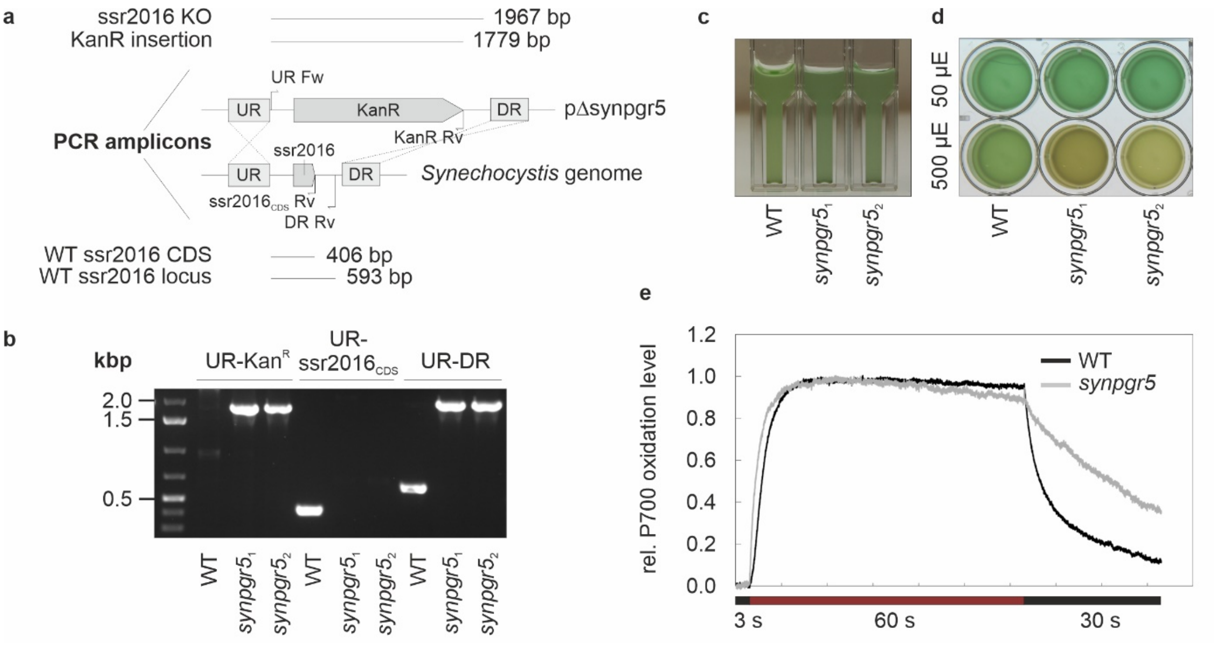

Supplementary Figure 6 Generation of an ssr2016 knockout mutant (*synpgr5*).

a,b Genomic replacement of the ssr2016 ORF by a KanR cassette and full segregation were confirmed by PCR. Genotyping primer binding sites and 5’-3’ orientation are indicated by labeled arrows; expected amplicon origins and sizes are indicated. Under low (**c**; 30 µE) to ambient light (**d**; 50 µE) no obvious deviation from WT cells was observed. However, enhanced susceptibility to high light intensities (**d**; 500 µE) and accelerated P700 oxidation/delayed P700 re-reduction upon on-/offset of FR light was observed (**e**), confirming the findings of former studies conferred with a gene interruption mutant of ssr2016 (SM2016)[5]. P700 oxidation curves (**e**) represent averages over all measurements of the respective genotypes represented in Fig. 1c.

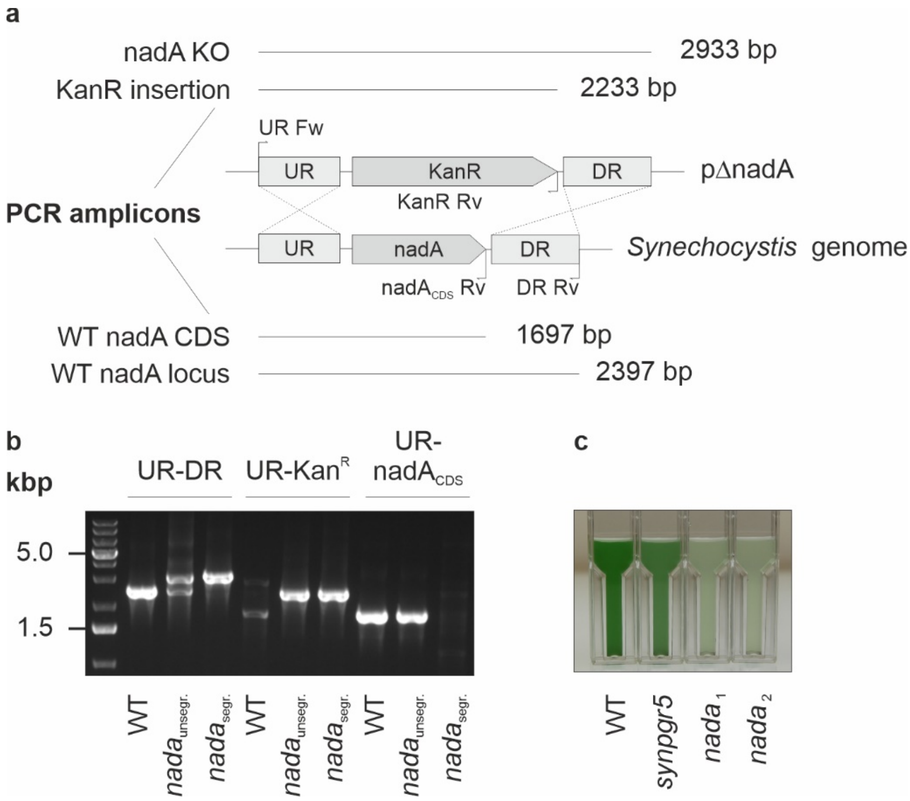

Supplementary Figure 7 Generation and initial phenotypical assessment of a *nada* knockout mutant.

a The genomic ORF encoding NadA (sll0622) was replaced with a KanR cassette by homologous recombination. **b** Segregation of the *nada* mutation was confirmed by PCR. Genotyping primer binding sites and 5’-3’ orientation are indicated by labeled arrows; expected amplicon origins and sizes are indicated. **c** Under low light (30 µE) obvious deviation from WT and *synpgr5* cells was observed in terms of pronounced paleness of *nada* cultures.

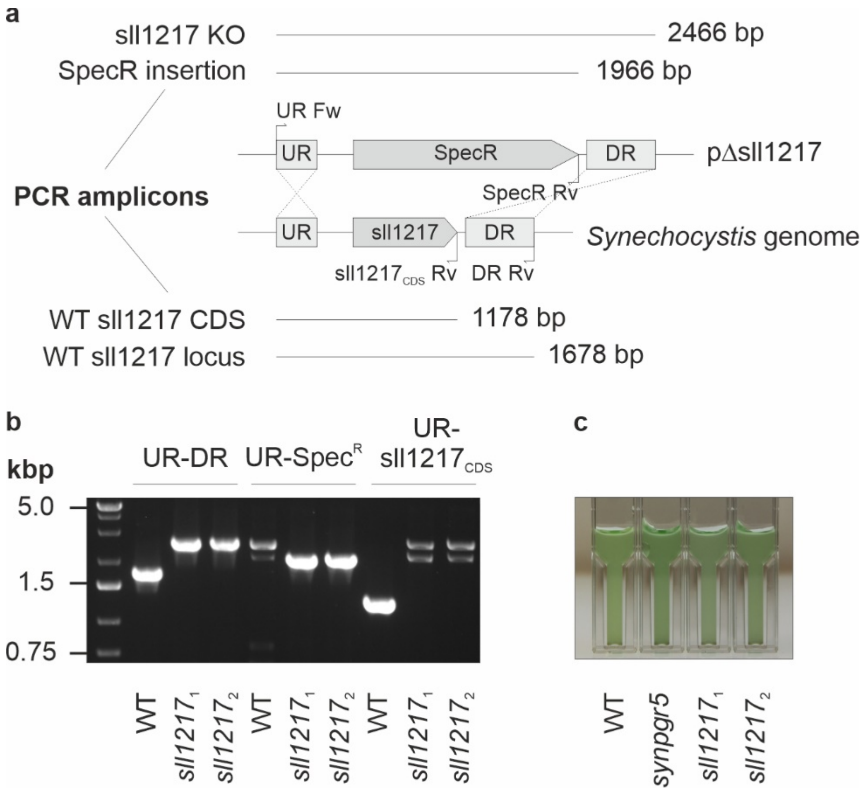

Supplementary Figure 8 Generation and initial phenotypical assessment of an *sll1217*-knockout mutant.

a The genomic ORF sll1217 was replaced with a SpecR cassette by homologous recombination. **b** Segregation of the *sll1217* mutation was confirmed by PCR. Genotyping primer binding sites and 5’-3’ orientation are indicated by labeled arrows; expected amplicon origins and sizes are indicated. **c** Under low light (30 µE) no obvious deviation from WT and *synpgr5* cells was observed.

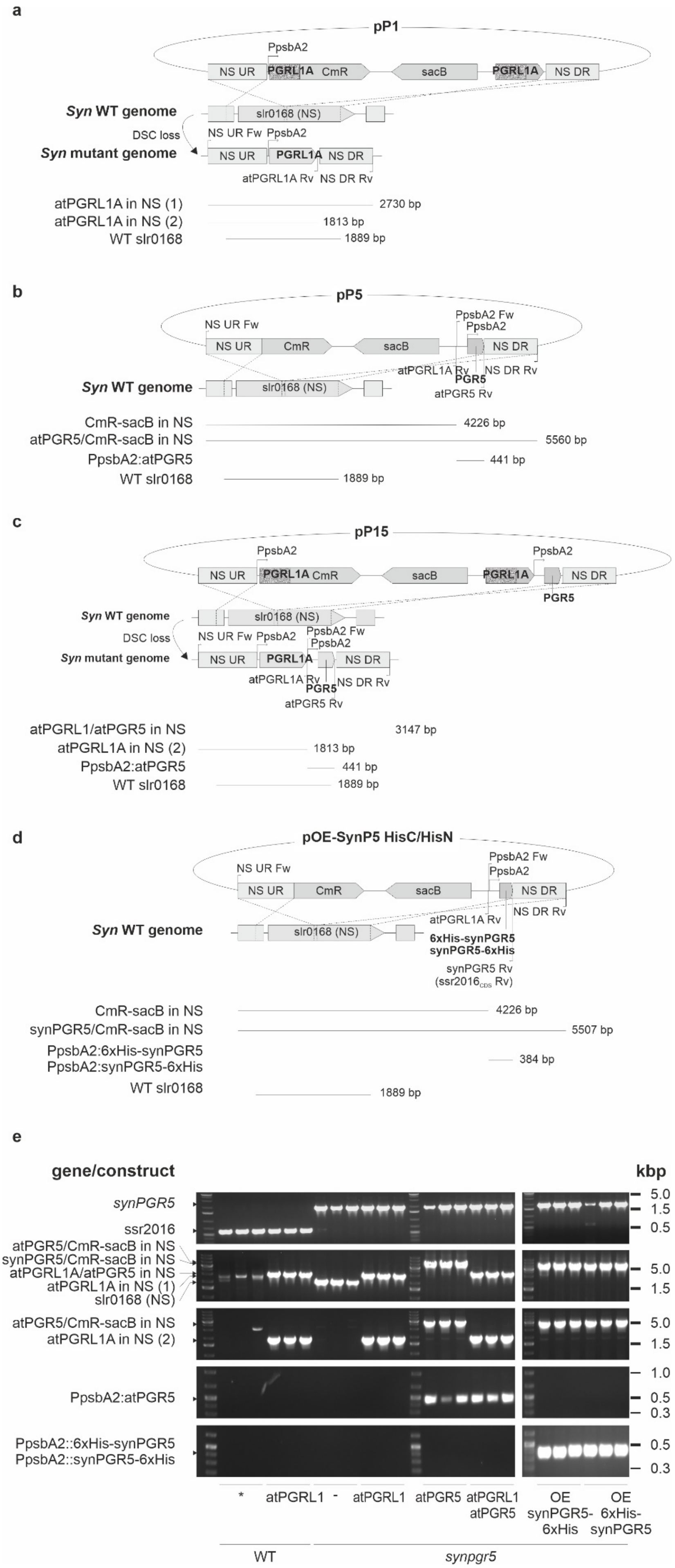
a
pP1
PpsbA2
NS UR
PGRL1A
CmR
sacB
PGRL1A
NS DR
Syn WT genome
slr0168 (NS)
DSC loss
NS UR Fw
PpsbA2
Syn mutant genome
NS UR
PGRL1A
NS DR
atPGRL1A Rv
NS DR Rv
atPGRL1A in NS (1)
2730 bp
atPGRL1A in NS (2)
1813 bp
WT slr0168
1889 bp
b
pP5
NS UR Fw
PpsbA2 Fw
PpsbA2
NS UR
CmR
sacB
NS DR
atPGRL1A Rv
NS DR Rv
Syn WT genome
slr0168 (NS)
PGR5
atPGR5 Rv
CmR-sacB in NS
4226 bp
atPGR5/CmR-sacB in NS
5560 bp
PpsbA2:atPGR5
441 bp
WT slr0168
1889 bp
c
pP15
PpsbA2
PpsbA2
NS UR
PGRL1A
CmR
sacB
PGRL1A
NS DR
PGR5
Syn WT genome
slr0168 (NS)
DSC loss
NS UR Fw
PpsbA2
PpsbA2 Fw
PpsbA2
Syn mutant genome
NS UR
PGRL1A
NS DR
atPGRL1A Rv
PGR5
NS DR Rv
atPGR5 Rv
atPGRL1/atPGR5 in NS
3147 bp
atPGRL1A in NS (2)
1813 bp
PpsbA2:atPGR5
441 bp
WT slr0168
1889 bp
d
pOE-SynP5 HisC/HisN
PpsbA2 Fw
NS UR Fw
PpsbA2
NS UR
CmR
sacB
NS DR
NS DR Rv
Syn WT genome
slr0168 (NS)
atPGRL1A Rv
6xHis-synPGR5
synPGR5-6xHis
synPGR5 Rv
(ssr2016CDS Rv)
CmR-sacB in NS
4226 bp
synPGR5/CmR-sacB in NS
5507 bp
PpsbA2:6xHis-synPGR5
PpsbA2:synPGR5-6xHis
384 bp
WT slr0168
1889 bp
e
gene/construct
kbp
synPGR5
ssr2016
atPGR5/CmR-sacB in NS
synPGR5/CmR-sacB in NS
atPGRL1A/atPGR5 in NS
atPGRL1A in NS (1)
slr0168 (NS)
atPGR5/CmR-sacB in NS
atPGRL1A in NS (2)
PpsbA2:atPGR5
PpsbA2::6xHis-synPGR5
PpsbA2::synPGR5-6xHis
5.0
1.5
0.5
5.0
1.5
5.0
1.5
1.0
0.5
0.3
0.5
0.3
*
atPGRL1
-
atPGRL1
atPGR5
atPGRL1
atPGR5
OE
synPGR5-
6xHis
OE
6xHis-
synPGR5
WT
synpgr5

Supplementary Figure 9 Generation of atPGR gene expression strains in *Synechocystis*. **a-d** Expression constructs were introduced into a genomic neutral site, ORF slr0168, by homologous recombination using a non-replicative vector as shuttle. When appropriate, chloramphenicol resistance (CmR) – sacB double selection cassettes were removed by negative selection on 5 % (w/v) sucrose agar as described earlier[6]. Redundant PGRL1A gene sequences for intrachromosomal recombination are displayed in grained grey. Genotyping primer binding sites and 5’-3’ orientation are indicated by labeled arrows; expected amplicon origins and sizes are indicated. **e** Genotyping PCR of three independent transformants each confirmed *synpgr5* genetic background, as well as transgene cassette presence and segregation status. Note that the WT control does not display an slr0168 amplicon of the expected size (2183 bp) because of complete deletion of the slr0168 CDS by replacement with a KanR selection cassette.

Supplementary Table 1 Primers used in this study.

Primer	genotyping short name	Supplementary Figure (Sup Fig.)	Sequence (5‘-3‘)
backbone_fwd			ACCTGGGCCCACTGCATCC
backbone_rev			GCTTACCTGTTTAAACTATCAGTG
ssr2016_UR_fwd	UR Fw	Sup Fig. 6	gatagtttaaacaggtaagcCCGGGTAATCCGGGTGGC
ssr2016_UR_rev			gggcgaacatggcagtgactCCTAAATTCCTACG
KanR_Cass_fwd	KanR Rv		caacgctcggttgccgccgggcgtttttTTTGGTCTCACGTTGGAATTC
KanR_Cass_rev			aaacgaagagGTAAAACAGCCAGCGCTG
ssr2016_DR_fwd			gctgttttacCTCTTCGTTTTCAATAATTCTTGCCAAAC
ssr2016_DR_rev	DR Rv		tggatgcagtgggcccaggtTTTCCACCGAAGGGCTGG
B2H ssr2016 Rv	*ssr2016_CDS Rv*		*tcagat**tctaga**TTAGGCCAATAAACCGAG*
nadA_UR_fwd	UR Fw	Sup. Fig. 7	acactgatagtttaaacaggtaagcGTTTACTGTCCCTGCTCC
nadA_UR_rev			acgtgagaccaaaTATGTTTCGGCTCCTGGAATATTTATAG
KanR_fwd			gagccgaaacataTTTGGTCTCACGTTGGAATTC
KanR_rev	KanR Rv		gattatgccacccGTAAAACAGCCAGCGCTG
nadA_DR_fwd			ctggctgttttacGGGTGGCATAATCAGGCTC
nadA_DR_rev	DR Rv		tggggtggatgcagtgggcccaggtTATTGCCACTAGAATTAGCCG
B2H sll0622 Rv	*nadA_CDS Rv*		*accacc**gaattc**CTAGGACATGGCCAGC*
sll1217_UR_fwd	UR Fw	Sup. Fig. 8	gatagtttaaacaggtaagcAGGACGGGGGGAAATTTC
sll1217_UR_rev			tgttcttctagagGGAAGAAACTGAGATAACTGATTG
SpecR_fwd			agtttcttccCTCTAGAAGAACAGCAAGGCCG
SpecR_rev	SpecR Rv		aagtctggaaGCCGCTCAATTCGCTGCG
sll1217_DR_fwd			attgagcggcTTCCAGACTTAAAATATTTATCACCTTTACTTC
sll1217_DR_rev	DR Rv		tggatgcagtgggcccaggtCCGCACAATGGTGTAGGG
B2H sll1217 Rv	*sll1217_CDS Rv*		*gtagtc**ggtacc**CTAGAAAGCAATTTCCGTTAAGCG*

Uppercase letter indicate specifically annealing sites; lowercase letters indicate complementary overhangs; red indicates not complementary sites and linear assembly was blunt-ligated; italics indicates B2H primer used for genotyping. The vector backbone was pICH69822

Supplementary References

1. Sugimoto, K., *et al.* A single amino acid alteration in PGR5 confers resistance to antimycin A in cyclic electron transport around PSI. *Plant Cell Physiol* **54**, 1525-1534 (2013).

2. Edgar, R. C. MUSCLE: multiple sequence alignment with high accuracy and high throughput. *Nucleic Acids Res* **32**, 1792-1797 (2004).

3. CyanoBase, http://genome.microbedb.jp/cyanobase.

4. Hertle, A. P., *et al.* PGRL1 is the elusive ferredoxin-plastoquinone reductase in photosynthetic cyclic electron flow. *Mol Cell* **49**, 511-523 (2013).

5. Yeremenko, N., *et al.* Open reading frame *ssr2016* is required for antimycin A-sensitive photosystem I-driven cyclic electron flow in the cyanobacterium *Synechocystis* sp. PCC 6803. *Plant Cell Physiol* **46**, 1433-1436 (2005).

6. Viola, S., Rühle T. & Leister D. A single vector-based strategy for marker-less gene replacement in *Synechocystis* sp. PCC 6803. *Microb Cell Fact* **13**, 4 (2014).
