# Supplementary material for: Evidence that cyanobacterial Sll1217 functions analogously to PGRL1 in enhancing PGR5-dependent cyclic electron flow
Source: Nat Commun. 2019 Nov 22;10:5299. doi: 10.1038/s41467-019-13223-0 (PMC6876563; doi:10.1038/s41467-019-13223-0)
Supplement: Supplementary file 2 — Reporting Summary [file 41467_2019_13223_MOESM2_ESM.pdf]

nature research

Corresponding author(s): Dario Leister

Last updated by author(s): Oct 25, 2019

Reporting Summary

Nature Research wishes to improve the reproducibility of the work that we publish. This form provides structure for consistency and transparency in reporting. For further information on Nature Research policies, see Authors & Referees and the Editorial Policy Checklist.

Statistics

For all statistical analyses, confirm that the following items are present in the figure legend, table legend, main text, or Methods section.

n/a	Confirmed	
☐	☒	The exact sample size (n) for each experimental group/condition, given as a discrete number and unit of measurement
☐	☒	A statement on whether measurements were taken from distinct samples or whether the same sample was measured repeatedly
☐	☒	The statistical test(s) used AND whether they are one- or two-sided<br>*Only common tests should be described solely by name; describe more complex techniques in the Methods section.*
☒	☐	A description of all covariates tested
☐	☒	A description of any assumptions or corrections, such as tests of normality and adjustment for multiple comparisons
☐	☒	A full description of the statistical parameters including central tendency (e.g. means) or other basic estimates (e.g. regression coefficient) AND variation (e.g. standard deviation) or associated estimates of uncertainty (e.g. confidence intervals)
☐	☒	For null hypothesis testing, the test statistic (e.g. F, t, r) with confidence intervals, effect sizes, degrees of freedom and P value noted<br>*Give P values as exact values whenever suitable.*
☒	☐	For Bayesian analysis, information on the choice of priors and Markov chain Monte Carlo settings
☒	☐	For hierarchical and complex designs, identification of the appropriate level for tests and full reporting of outcomes
☒	☐	Estimates of effect sizes (e.g. Cohen's d, Pearson's r), indicating how they were calculated

Our web collection on statistics for biologists contains articles on many of the points above.

Software and code

Policy information about availability of computer code

Data collection	n.a.
Data analysis	n.a.

For manuscripts utilizing custom algorithms or software that are central to the research but not yet described in published literature, software must be made available to editors/reviewers. We strongly encourage code deposition in a community repository (e.g. GitHub). See the Nature Research guidelines for submitting code & software for further information.

Data

Policy information about availability of data

All manuscripts must include a data availability statement. This statement should provide the following information, where applicable:

- Accession codes, unique identifiers, or web links for publicly available datasets
- A list of figures that have associated raw data
- A description of any restrictions on data availability

Additional data that supports the findings of this study are available from the corresponding author upon reasonable request.

Field-specific reporting

Please select the one below that is the best fit for your research. If you are not sure, read the appropriate sections before making your selection.

☒ Life sciences ☐ Behavioural & social sciences ☐ Ecological, evolutionary & environmental sciences

For a reference copy of the document with all sections, see nature.com/documents/nr-reporting-summary-flat.pdf

Life sciences study design

All studies must disclose on these points even when the disclosure is negative.

Sample size	Sample size for P700 PAM measurements was dictated by the time point at which the maximum amount of cultures simultaneously had reached a growth stage suitable for measurement, and the number of according cultures per genotype having reached/not surpassed this point. With cultivation room and media batch size being practical limiting factors, the sample sizes presented are the maximum we could obtain in the context of a single cultivation batch. The presented sample size is regarded sufficiently representative for reasons of the employed working system being prokaryotic, and performed measurements of 2 ml OD5 cells yielding integrals over the physiological state of >10^8 cells for each data point, respectively.<br><br>Sample size on n=3 independently transformed clones for B2H interaction assays was chosen according to convention and due to high reproducibility of results.
Data exclusions	n.a.
Replication	Subsets of the entire mutant set presented were measured in the same manner as described repeatedly before the presented study. Qualitatively, results were found to be highly robust. However, quantitative deviations of P700 oxidation half times of identical genotypes/clones were observed among batches, probably resulting from a mixture of (i) slight inconsistencies between media batches, (ii) differences in age/vitality of plate cultures used as inoculum, and, apparently, (ii) outside climate. Hence, we decided to acquire to-be-shown data from a single batch of media and antibiotics, and cultivate all assayed cell material simultaneously to optimize data signal-to-noise ratio.
Randomization	Synechocystis cultures were distributed randomly on shakers during cultivation stage, and assigned random numbers prior to preparation for PAM measurements. Measurements were conducted in ascending order of assigned numbers, yielding randomized running order of physiological measurements.
Blinding	Culture identity was only determined after measurements and subsequent protein extraction were performed, rendering further blinding unnecessary.

Reporting for specific materials, systems and methods

We require information from authors about some types of materials, experimental systems and methods used in many studies. Here, indicate whether each material, system or method listed is relevant to your study. If you are not sure if a list item applies to your research, read the appropriate section before selecting a response.

Materials & experimental systems

n/a	Involved in the study	
☐	☒	Antibodies
☒	☐	Eukaryotic cell lines
☒	☐	Palaeontology
☒	☐	Animals and other organisms
☒	☐	Human research participants
☒	☐	Clinical data

Methods

n/a	Involved in the study	
☒	☐	ChIP-seq
☒	☐	Flow cytometry
☒	☐	MRI-based neuroimaging

Antibodies

Antibodies used	Anti-AtPGR5, Anti-AtPGRL1, Goat Anti-Rabbit IgG Antibody HRP-conjugate (Sigma-Aldrich A9169); all antibodies have been used in dilutions of 1:10000 in 1xTBST with 3 % (w/v) BSA.
Validation	Anti-PGR5 has been described by Munekage et al. (2002, Cell); Anti-PGRL1 has been described by DalCorso et al. (2008, Cell)
